# Effects of 100 years wastewater irrigation on resistance genes, class 1 integrons and IncP-1 plasmids in Mexican soil

**DOI:** 10.3389/fmicb.2015.00163

**Published:** 2015-03-03

**Authors:** Sven Jechalke, Melanie Broszat, Friederike Lang, Christina Siebe, Kornelia Smalla, Elisabeth Grohmann

**Affiliations:** ^1^Institute for Epidemiology and Pathogen Diagnostics, Julius Kühn-Institut – Federal Research Centre for Cultivated Plants (JKI)Braunschweig, Germany; ^2^Department of Infectious Diseases, University Hospital FreiburgFreiburg, Germany; ^3^Microbiology, Faculty for Biology, Albert-Ludwigs-University FreiburgFreiburg, Germany; ^4^Chair of Soil Ecology, Albert-Ludwigs-University FreiburgFreiburg, Germany; ^5^Instituto de Geología, Universidad Nacional Autónoma de México, Ciudad UniversitariaMexico City, Mexico

**Keywords:** wastewater irrigation, IncP-1 plasmids, class 1 integrons, quaternary ammonium compound resistance, tetracycline resistance, aminoglycoside resistance

## Abstract

Long-term irrigation with untreated wastewater can lead to an accumulation of antibiotic substances and antibiotic resistance genes in soil. However, little is known so far about effects of wastewater, applied for decades, on the abundance of IncP-1 plasmids and class 1 integrons which may contribute to the accumulation and spread of resistance genes in the environment, and their correlation with heavy metal concentrations. Therefore, a chronosequence of soils that were irrigated with wastewater from 0 to 100 years was sampled in the Mezquital Valley in Mexico in the dry season. The total community DNA was extracted and the absolute and relative abundance (relative to 16S rRNA genes) of antibiotic resistance genes (*tet*(W), *tet*(Q), *aadA*), class 1 integrons (*intI1*), quaternary ammonium compound resistance genes (*qacE*+*qacEΔ1*) and IncP-1 plasmids (*korB*) were quantified by real-time PCR. Except for *intI1* and *qacE*+*qacEΔ1* the abundances of selected genes were below the detection limit in non-irrigated soil. Confirming the results of a previous study, the absolute abundance of 16S rRNA genes in the samples increased significantly over time (linear regression model, *p* < 0.05) suggesting an increase in bacterial biomass due to repeated irrigation with wastewater. Correspondingly, all tested antibiotic resistance genes as well as *intI1* and *korB* significantly increased in abundance over the period of 100 years of irrigation. In parallel, concentrations of the heavy metals Zn, Cu, Pb, Ni, and Cr significantly increased. However, no significant positive correlations were observed between the relative abundance of selected genes and years of irrigation, indicating no enrichment in the soil bacterial community due to repeated wastewater irrigation or due to a potential co-selection by increasing concentrations of heavy metals.

## Introduction

Wastewater irrigation is a widely used practice worldwide, especially in arid and semiarid regions, to alleviate water shortages in agriculture (Siebe and Cifuentes, 1995; Jimenez and Chávez, 2004; Elifantz et al., 2011; Frenk et al., 2014). More than 20 million ha of land are estimated to be irrigated with wastewater globally, and particularly in developing countries the number of people consuming produce irrigated with poorly or non-treated water is increasing (Amoah et al., 2007; Raschid-Sally and Priyantha, 2008). Since wastewater irrigation provides nutrients that improve plant growth, reduces the need for fertilizer application, and increases the productivity of soils with poor fertility, it is expected to expand further (Gatica and Cytryn, 2013). An example is the Mezquital Valley, where untreated wastewater mixed with surface run-off released from the Mexico City Metropolitan area, located 80 km south of the valley, has been used since more than 100 years (Raschid-Sally and Priyantha, 2008; Dalkmann et al., 2012).

However, besides high concentrations of organic matter, wastewater typically contains large amounts of pollutants including detergents, heavy metals, pharmaceuticals including antibiotics, as well as pathogenic and antibiotic resistant bacteria carrying resistance determinants, class 1 integrons and mobile genetic elements (MGEs) (Baquero et al., 2008; Levantesi et al., 2010; Moura et al., 2010; Chávez et al., 2011; Malik and Aleem, 2011; Bruchmann et al., 2013; Rizzo et al., 2013; Manzetti and Ghisi, 2014). The coexistence of pathogens, antibiotic resistance genes, antibiotics, and heavy metals raises concerns about antibiotic resistance genes being mobilized, propagated, and ultimately transferred to bacteria that are pathogenic to humans (Baquero et al., 2009; Canton, 2009; Wright, 2010). Previous studies showed an increase of pharmaceuticals (Kinney et al., 2006; Ternes et al., 2007; Chen et al., 2011; Tamtam et al., 2011; Dalkmann et al., 2012), resistance determinants, and antibiotic resistant bacteria in soils due to wastewater-irrigation (Dalkmann et al., 2012; Broszat et al., 2014; Chen et al., 2014).

In soils from the Mezquital Valley, long-term wastewater irrigation led to an increase in the abundance of *sul* genes, encoding resistance toward sulfonamides, and an accumulation of antibiotics. The sulfonamide resistance gene *sul1* is often associated with the *qacEΔ1* gene on class 1 integrons, especially in clinical settings (Wellington et al., 2013; Gillings, 2014). However, class 1 integrons are also widely disseminated in environmental settings such as soil, sewage sludge and animal slurries, and are able to acquire, exchange, and express genes embedded in gene cassettes, which can include resistance genes for almost all antibiotic families (Moura et al., 2010; Gaze et al., 2011; Stalder et al., 2012; Gillings, 2014; Jechalke et al., 2014b). For example, it was observed that class 1 integrons from manure and manured soil frequently carried *aadA* gene cassettes, conferring resistance toward streptomycin and spectinomycin (Binh et al., 2009; Heuer et al., 2012). Exposure to antibiotics can up-regulate the *intI1* expression by triggering the SOS response and ultimately increase gene cassette recombination rates (Cambray et al., 2011; Hocquet et al., 2012) and might also lead to co-selection of antibiotic resistance, e.g., by heavy metals (Rosewarne et al., 2010). Furthermore, class 1 integrons are often located on MGEs such as transposons and plasmids, e.g., of the IncP-1ε incompatibility group, facilitating their transfer and spread within bacterial communities (Stokes and Gillings, 2011; Heuer et al., 2012; Gillings, 2014). Hence, due to their ability to foster bacterial adaptation to environmental perturbation, class 1 integrons might be used as a universal marker for selective pressure in the environment. However, knowledge of long-term effects of wastewater application containing antibiotics and heavy metals on the abundance of class 1 integrons, related antibiotic resistance genes and MGEs is scarce.

An interesting study site to explore such long-term effects of human impact is located in the Mezquital Valley, where long-term untreated wastewater irrigation for up to 100 years has increased soil organic matter contents as well as nitrogen and phosphorous concentrations up to 2-fold (Siebe, 1998), which in turn resulted in an increase of total soil microbial biomass (Friedel et al., 2000). Also total heavy metal contents including Zn, Cu, Cd, Pb, and Cr as well as bioavailable concentrations of Zn, Cu, and Cd increased with increasing duration of irrigation between 3- and 9-fold in relation to non-irrigated soils (Gutiérrez-Ruiz et al., 1995; Siebe, 1995; Siebe and Cifuentes, 1995). In the present study, the effects of long-term wastewater application on the abundance of IncP-1 plasmids, class 1 integrons and their typically associated resistance genes (*qacEΔ1, aadA*), as well as municipal wastewater associated resistance genes *tet*(W) and *tet*(Q) (Auerbach et al., 2007; Storteboom et al., 2010; Burch et al., 2014) were analyzed in soil from Mezquital Valley. Additionally, concentrations of heavy metals and other inorganic substances were determined and correlated with the duration of irrigation and with the abundance of the selected genes.

## Materials and methods

### Soil samples

The sites of the irrigation chronosequence and the soil sampling were described by Dalkmann et al. (2012). Briefly, sites were selected on behalf of duration of irrigation with untreated wastewater (0, 1.5, 3, 6, 8, 85, and 100 years of irrigation) and soil type. Soils in the Mezquital Valley have been classified as Leptosols, Vertisols, and Phaeozems (Siebe, 1998). In all selected fields water is provided by the same main distribution channel, and each field is irrigated about 10–12 times every year by overflow. Each single irrigation event lasts 6 to 24 h according to the field size. The discharges from Mexico City are dominated by domestic sewage (65%), followed by discharges of the service sector (20%) and the industry (15%). The travel distance to the valley is 80 km, along which the sewage is partly homogenized and large suspended particles and floating materials are removed by sedimentation or retained by bar screens (Gutiérrez-Ruiz et al., 1995). Although water quality is variable in time, we assume that frequent and long lasting irrigations produce similar pollutant loads in all fields over time.

All fields analyzed here were sampled during the dry season. Each individual field was subdivided into four parcels, two on the wastewater inflow side and two on the wastewater outflow side of the field. Samples were taken from each of the parcels by combining 12 subsamples taken with an auger at a depth of 0–30 cm. Soil samples were transported in plastic bags at 4°C and stored at −21°C until further processing.

### Extraction of total community DNA and quantification of target genes

Total community (TC-) DNA was extracted from 0.5 g soil using the NucleoSpin® Soil Kit according to the manufacturer's protocol (Macherey-Nagel, Düren, Germany). DNA extracted from the four parcels was combined and target genes were quantified in triplicate by quantitative real-time PCR 5′-nuclease assays (qPCR) in a CFX96 real-time PCR detection system (Bio-Rad, Hercules, CA) as previously described for *korB* of IncP-1 plasmids (Jechalke et al., 2013), class 1 integron integrase gene *intI1* (Barraud et al., 2010), quaternary ammonium compound resistance genes *qacE* and the *qacEΔ1* variant (Jechalke et al., 2014b), tetracycline resistance gene *tet*(W) (Smith et al., 2004), and the streptomycin resistance gene *aadA* (Walsh et al., 2011), which was frequently found on class 1 integrons (Moura et al., 2007; Schlüter et al., 2007; Binh et al., 2009; Gaze et al., 2011). The 16S rRNA gene (*rrn*) copies were quantified using the primers BACT1369F and PROK1492R and the probe TM1389F (Suzuki et al., 2000). To adjust for differences in bacterial DNA and amplification efficiency between samples, target numbers of the respective genes were divided by the *rrn* copy numbers and the results were log transformed. The Pearson product-moment correlations between target gene copy number/relative abundance and years of irrigation were tested using the CORR procedure of the SAS statistical package (*p* < 0.05; SAS 9.3; SAS Institute Inc., Cary, NC). Multiple comparisons of means were performed using the GLIMMIX procedure (Tukey test, *p* < 0.05; SAS 9.3).

### Elemental analysis of soil samples

Soil samples were air-dried (<40°C), sieved to pass a 2 mm nylon mesh, and homogenized using a disc mill with agate stone beakers. Afterwards, 200 mg of homogenized and dried (105°C) samples were placed in clean Teflon vessels and 4 ml of HNO_3_ (65%, Suprapur, Merck KGaA, Darmstadt, Germany) and 2 ml of H_2_O_2_ (35%, Suprapur, Merck KGaA) were added. After 12 h of pre-hydrolysis vessels were locked and subjected to 30 min of microwave treatment (MLS 1200 mega microwave, MLS GmbH, Leutkirch, Germany) with a maximum power of 600 W and a maximum temperature of 110°C. After digestion, samples were quantitatively transferred to volumetric flasks, filled up to 100 ml, and afterwards stored in polythene containers. Analyses of total concentrations of Zn, Cu, Cd, Pb, Ni, Cr, Mn, S, and P were performed by Inductive Coupled Plasma Atomic Emission Spectroscopy (ICP-AES, site-on plasma; Spectro CIROS CCD, SPECTRO Analytical Instruments, Kleve, Germany) from soil samples of years 0, 1.5, 3, 6, 8, 85, and 100. All samples were digested and analyzed in duplicate and concentrations were expressed on dry weight basis.

Potentially bioaccessible concentrations of Zn, Cu, and Cd were analyzed in 1 M NH_4_NO_3_ extracts (soil:solution ratio 1:2.5) by graphite furnace atomic absorption spectroscopy (Schöning and Brümmer, 2008) from samples of years 0, 3, 8, 85, and 100 (see above). The soil pH values from samples of all years were measured potentiometrically in a suspension of 0.01 M CaCl_2_ solution (soil:solution ratio 1:2.5 wt/vol), and the electric conductivities in suspensions with distilled water (soil:water ratio 1:2.5 wt/vol). Total organic carbon (TOC) was quantified in a CHNS/O analyzer (Perkin Elmer 2400 Series II, PerkinElmer, Waltham, MA, USA). Bioaccessible P in samples of years 0, 8, 85, and 100 was quantified colorimetrically in 1 M NaHCO_3_ extracts (Schlichting et al., 1995).

The Pearson product-moment correlations between the concentrations of metals, target genes and the years of irrigation were tested using the CORR procedure of the SAS statistical package (*p* < 0.05; SAS 9.3; SAS Institute Inc., Cary, NC).

## Results

### Quantification of target genes

**Figure 1:**
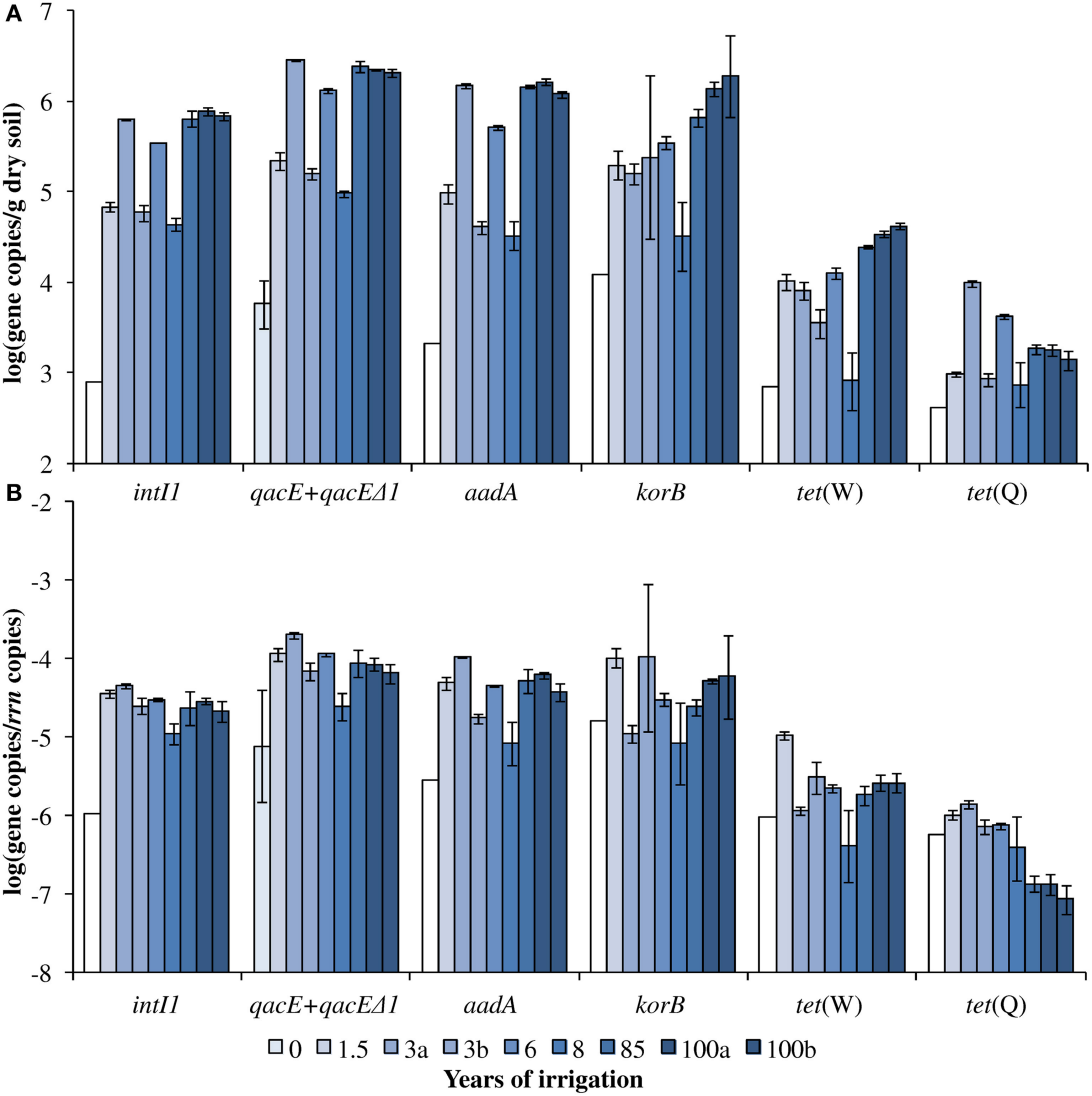
**Dry season (A) absolute and (B) relative gene abundance over the period of 100 years of irrigation**. Bars and error bars indicate means and respective standard deviations of three technical replicates of one DNA extract from one field, respectively. Fields irrigated for the same period of time are differentiated by small letters (a, b). Except for *qacE*+*qacEΔ1* absolute and relative abundance (indicated by light blue bars), the year 0 values indicate the detection limits for the respective genes (white bars). The detection limits for the relative abundances were calculated based on the mean 16S rRNA gene copy number of year 0 and the detection limits of the respective genes.

The abundance of *rrn* copies per gram soil (dry weight) was quantified and revealed similar results as observed in the previous study by Dalkmann et al. (2012) for the same samples. A significant positive correlation was observed between the *rrn* copy number and years of irrigation (0–100 years, *p* < 0.0001, Pearson correlation coefficient 0.75; 1.5–100 years, *p* < 0.0001, Pearson correlation coefficient 0.79), which coincides with previous findings of an increased microbial biomass in long-term irrigated soils (Friedel et al., 2000). Samples including the period of 1.5–100 years were selected for the analysis of target genes (Figure 1) due to a low abundance in the samples not irrigated with wastewater (0 years), which was predominantly below detection limit of the qPCR method (between log 2–3 gene copies/g dry soil). Significant correlations between gene abundance (per gram dry soil) and years of irrigation were observed for *intI1, qacE*+*qacEΔ1, aadA, korB*, and *tet*(W), while no significant correlation was observed for *tet*(Q) (Table 1). On the contrary, regarding the abundance of genes relative to *rrn* copies, no significant correlations were found between years of irrigation and *intI1, qacE*+*qacEΔ1, aadA, korB*, and *tet*(W), while a significant negative correlation was found for *tet*(Q). The abundance of *qacE* and *qacEΔ1* genes in irrigated soils on average was three times higher than of *intI1* (Tukey test, *p* < 0.05), while the gene abundances of *korB, intI1*, and *aadA* were not significantly different. The mean abundances of *tet*(W) and *tet*(Q) were about 1 and 2 orders of magnitude lower than the abundance of *korB, intI1*, and *aadA*, respectively.

**Table 1:** **Correlation analysis of absolute (log(gene copies/g dry soil)) and relative (log(gene copies/*rrn* copies)) gene abundances in soil samples from the dry season and the duration of wastewater irrigation (1.5–100 years of irrigation; Pearson correlation, *p* < 0.05; significance indicated by asterisk)**.

	**Absolute abundance**		**Relative abundance**	
	**PCC**	***p*-value**	**PCC**	***p*-value**
*intI1*	0.68	0.0002*	−0.13	0.55
*qacE+qacEΔ1*	0.61	0.002*	−0.1	0.65
*aadA*	0.66	0.0005*	0.24	0.26
*korB*	0.71	0.0001*	0.12	0.58
*tet*(W)	0.73	<0.0001*	0.05	0.83
*tet*(Q)	−0.09	0.66	−0.9	<0.0001*

*PCC, Pearson correlation coefficient*.

### Concentrations of inorganic compounds

Total (acid extractable) and potentially bioaccessible (NH_4_NO_3_-extractable) concentrations of selected metals were analyzed in dry season samples and ranged from 0 until 0.8 mg/g soil (Table 2, Tables S1, S2). Additionally, total concentrations of P and S in soil samples increased with the length of irrigation.

**Table 2:** **Correlation of metals and inorganic compounds with the duration of wastewater irrigation (0–100 years of irrigation; Pearson correlation, *p* < 0.05; significance indicated by asterisk)**.

**Fraction**	**Compound**	**min [mg/g]**	**max [mg/g]**	**PCC**	***p*-value**
Total	Zn	0.046	0.281	0.95	<0.0001*
	Cu	0.012	0.07	0.97	<0.0001*
	Cd	n.d.	n.d.	n.d.	n.d.
	Pb	n.d.	0.063	0.82	0.004*
	Ni	0.018	0.043	0.92	0.0002*
	Cr	0.035	0.099	0.85	0.002*
	Mn	0.5	0.8	−0.73	0.02*
	P	0.3	1.9	0.91	0.0002*
	S	0.2	1.1	0.8	0.005*
Bioaccessible	Zn	0.02 μg/g	0.084 μg/g	0.94	0.0016*
	Cu	0.01 μg/g	0.11 μg/g	0.94	0.0016*
	Cd	0.0001 μg/g	7 ng/g	0.94	0.002*
	P	0.033 mg/g	0.08 mg/g	−0.51	0.38

*PCC, Pearson correlation coefficient; n.d., not detected. Additionally, minimum and maximum concentrations within the chronosequence are shown*.

Total soil organic carbon contents also increased significantly with the duration of irrigation (0–100 years irrigation, *p* = 0.0013, Pearson correlation coefficient 0.89), while soil pH, electrical conductivity, and plant available P contents are not significantly correlated with the years of irrigation.

### Correlation of gene abundances and environmental variables

Absolute and relative abundances of genes *intI1, qacE*+*qacEΔ1, aadA, korB, tet*(W), and *tet*(Q) were correlated with total concentrations of heavy metals Zn, Cu, Pb, Ni, Cr, and Mn as well as with total concentrations of P and S (Table 3). While for the relative abundance of genes only *tet*(Q) showed significant negative correlations to Zn, Cu, Pb, Ni, Cr, and P, absolute abundances of *korB* and *tet*(W) showed positive correlations to Zn, Cu, Pb, Ni, Cr (only *korB*), P, and S, as well as negative correlations to Mn (Table 3). The concentration of sulfur was positively correlated with absolute abundances of *intI1, qacE*+*qacEΔ1, aadA, korB*, and *tet*(W), but not with *tet*(Q).

**Table 3:** **Correlation of absolute (log(gene copies/g soil)) and relative abundance (log(gene copies/*rrn* copies)) of target genes with concentrations of heavy metals, S, and P (1.5–100 years of irrigation; Pearson correlation, *p* < 0.05; significance indicated in bold and by asterisk)**.

		**Zn**	**Cu**	**Pb**	**Ni**	**Cr**	**Mn**	**P**	**S**
Absolute abundance	*intI1*	0.67	0.66	0.68	0.65	0.58	−0.52	0.68	**0**.**82**
		0.071	0.074	0.062	0.084	0.132	0.183	0.062	**0**.013*
	*qacE+qacEΔ1*	0.61	0.60	0.65	0.59	0.54	−0.48	0.62	**0**.**78**
		0.110	0.120	0.082	0.123	0.167	0.224	0.104	**0**.022*
	*aadA*	0.64	0.64	0.65	0.62	0.56	−0.56	0.64	**0**.**81**
		0.085	0.089	0.080	0.101	0.146	0.153	0.086	**0**.014*
	*korB*	**0**.**81**	**0**.**80**	**0**.**76**	**0**.**78**	**0**.**72**	−**0**.**74**	**0**.**89**	**0**.**74**
		**0**.014*	**0**.016*	**0**.028*	**0**.023*	**0**.042*	**0**.037*	**0**.003*	**0**.038*
	*tet*(W)	**0**.**75**	**0**.**74**	**0**.**74**	**0**.**71**	0.69	−**0**.**77**	**0**.**78**	**0**.**75**
		**0**.032*	**0**.038*	**0**.038*	**0**.046*	0.059	**0**.027*	**0**.024*	**0**.033*
	*tet*(Q)	−0.08	−0.10	0.08	−0.08	−0.10	0.02	−0.02	0.33
		0.845	0.807	0.855	0.847	0.806	0.966	0.963	0.423
Relative abundance	*intI1*	−0.14	−0.17	−0.12	−0.18	−0.16	−0.36	0.04	0.25
		0.738	0.681	0.776	0.669	0.702	0.386	0.932	0.550
	*qacE+qacEΔ1*	−0.07	−0.11	0.01	−0.09	−0.05	−0.28	0.04	0.29
		0.875	0.804	0.982	0.840	0.898	0.496	0.920	0.486
	*aadA*	0.25	0.23	0.25	0.21	0.21	−0.55	0.31	0.58
		0.552	0.587	0.552	0.617	0.626	0.159	0.447	0.136
	*korB*	0.17	0.15	0.09	0.14	0.16	−0.48	0.34	0.04
		0.679	0.715	0.827	0.748	0.705	0.231	0.405	0.929
	*tet*(W)	0.09	0.06	0.06	0.05	0.11	−0.54	0.19	0.06
		0.828	0.883	0.894	0.901	0.789	0.171	0.656	0.891
	*tet*(Q)	**−0.91**	**−0.93**	**−0.79**	**−0.90**	**−0.83**	0.49	**−0.81**	−0.59
		0.002*	0.001*	0.020*	0.002*	0.010*	0.214	0.016*	0.120

*Upper number = Pearson correlation coefficient (PCC); lower number = p-value*.

Correlations between absolute and relative abundances of genes *intI1, qacE*+*qacEΔ1, aadA, korB, tet*(W), and *tet*(Q) and potentially bioaccessible concentrations of heavy metals Zn, Cu, and Cd, as well as with soil electrical conductivity, pH, and TOC were also analyzed (Table 4). Similar to the total concentrations, only the relative abundance of *tet*(Q) showed a significant correlation to bioaccessible concentrations of Zn, Cu, and Cd, but also to TOC. For the absolute abundance of genes only *korB* and *tet*(W) were correlated to the bioaccessible concentrations of Cu. Except for *tet*(Q) all absolute abundances of tested genes were positively correlated to TOC concentrations. The electrical conductivity showed a significant positive correlation to *tet*(Q) absolute abundance only. No significant correlations were observed between gene abundances and soil pH.

**Table 4:** **Correlation of absolute (log(gene copies/g soil)) and relative abundance (log(gene copies/*rrn* copies)) of target genes with bioaccessible concentrations of heavy metals as well as with electrical conductivity (E.c.), pH and total organic carbon (TOC) (1.5–100 years of irrigation; Pearson correlation, *p* < 0.05; significance indicated in bold and by asterisk)**.

		**Zn**	**Cu**	**Cd**	**E.c.**	**pH**	**TOC**
Absolute abundance	*intI1*	0.57	0.80	0.66	0.33	0.48	**0.81**
		0.237	0.055	0.154	0.429	0.229	0.016*
	*qacE+qacEΔ1*	0.50	0.76	0.61	0.39	0.45	**0.74**
		0.310	0.080	0.195	0.346	0.259	0.037*
	*aadA*	0.56	0.78	0.65	0.36	0.48	**0.77**
		0.253	0.066	0.163	0.381	0.226	0.027*
	*korB*	0.73	**0.85**	0.75	−0.22	0.38	**0.85**
		0.101	0.032*	0.084	0.605	0.347	0.008*
	*tet*(W)	0.72	**0.90**	0.78	−0.04	0.47	**0.77**
		0.108	0.016*	0.066	0.919	0.235	0.026*
	*tet*(Q)	−0.21	0.09	−0.07	**0.78**	0.25	0.19
		0.694	0.861	0.895	0.022*	0.548	0.657
Relative abundance	*intI1*	−0.11	0.13	0.01	0.56	0.01	0.07
		0.833	0.805	0.986	0.151	0.986	0.861
	*qacE+qacEΔ1*	−0.07	0.22	0.09	0.60	0.07	0.10
		0.895	0.676	0.867	0.117	0.878	0.819
	*aadA*	0.26	0.51	0.38	0.55	0.28	0.41
		0.625	0.305	0.459	0.161	0.505	0.318
	*korB*	0.29	0.27	0.26	−0.49	−0.10	0.13
		0.579	0.601	0.618	0.221	0.809	0.757
	*tet*(W)	0.38	0.48	0.42	−0.26	0.02	0.03
		0.459	0.337	0.403	0.538	0.959	0.951
	*tet*(Q)	**−0.92**	**−0.85**	**−0.86**	0.48	−0.35	**−0.77**
		0.009*	0.033*	0.027*	0.231	0.389	0.027*

*Upper number = Pearson correlation coefficient (PCC); lower number = p-value*.

## Discussion

Wastewater typically contains a diverse mixture of pharmaceuticals, pathogenic bacteria, antibiotic resistant bacteria, resistance genes, and heavy metals, which can reach and affect the environment and might pose a risk for human health when wastewater is not properly treated or directly applied as fertilizer (Moura et al., 2010; Bouki et al., 2013; Norton-Brandao et al., 2013; Rivera-Utrilla et al., 2013). In the Mezquital Valley, results from a cross-sectional survey done 20 years ago revealed that intestinal helminth infections represent the highest risk associated with exposure to wastewater irrigation (Blumenthal et al., 1991; Cifuentes et al., 1991). However, no recent epidemiological data exist on the prevalence of disease in the region with respect to wastewater irrigation. Also, little is known worldwide about long-term effects of wastewater application on the accumulation of heavy metals and effects on the abundance of resistance genes and associated MGE in agricultural soil. Therefore, a chronosequence of soils was sampled from the Mezquital Valley that was irrigated with wastewater from zero up to 100 years.

A previous study using the same samples had demonstrated that irrigation with wastewater over several decades led to an accumulation of several pharmaceuticals in soil, such as ciprofloxacin, sulfamethoxazole, and carbamazepine (Dalkmann et al., 2012). At the same time, long-term irrigation with waste-water led to a reduced sorption of sulfamethoxazole, while the sorption of ciprofloxacin was not affected (Dalkmann et al., 2014). Furthermore, wastewater application was correlated with an increase in organic carbon, total microbial biomass and activity accompanied by an increase in absolute numbers of *sul1* and *sul2* genes, conferring resistance to sulfonamide antibiotics such as sulfamethoxazole (Siebe and Fischer, 1996; Friedel et al., 2000; Dalkmann et al., 2012). However, the abundance of *sul* genes relative to 16S rRNA genes did not correlate with the duration of irrigation (Dalkmann et al., 2012), indicating no enrichment of sulfonamide resistant populations within the soil bacterial community.

In this study we observed, besides the increase in absolute abundance of *rrn* copies, which was already demonstrated by Dalkmann et al. (2012) using the same soil samples, an increase in absolute abundance of *intI1, qacE*+*qacEΔ1, aadA, korB*, and *tet*(W) genes with the duration of irrigation (Table 1). No significant increase was observed for *tet*(Q) which was detected in very low abundance. Additionally, confirming the results of previous studies (Siebe, 1994; Siebe and Cifuentes, 1995; Guedron et al., 2014), we could show that the concentrations of the heavy metals Zn, Cu, Pb, Ni, and Cr increased with the duration of irrigation, while Mn decreased and Cd was below detection limit (Table 2). The decrease of Mn in the soils is coherent with the frequent flooding with large amounts of water, which induces temporal reducing conditions in the soil under which Mn is mobilized and lixiviated with the percolating water as Mn^2+^ (Siebe and Fischer, 1996). Additionally, NH_4_NO_3_-extractable concentrations of Zn, Cu, and Cd, which are considered to represent the bioaccessible fraction due to the gentle extraction, increased with the duration of irrigation (Siebe, 1994).

Similar to antibiotics, which interact with the soil solid phase in sorption and desorption reactions controlling their biotransformation and biological effects (Jechalke et al., 2014a), metals are not readily degraded and thus can affect bacterial populations for extended periods (Stepanauskas et al., 2005). Genes encoding metal resistance determinants are frequently found on MGEs such as transposons and plasmids, which also carry integrons and antibiotic resistance genes (Baker-Austin et al., 2006). Thus, the exposure of bacterial communities to metals can select for metal-resistant strains but also indirectly for bacteria resistant to unrelated toxicants, such as antibiotics, by co-selection processes (Stepanauskas et al., 2005; Baker-Austin et al., 2006). Consequently, a number of studies comparing contaminated and reference sites observed direct or indirect associations between the presence of metals and elevated antibiotic resistance and abundance of class 1 integrons (Baker-Austin et al., 2006; Wright et al., 2008; Rosewarne et al., 2010). Class 1 integrons, which are able to acquire, exchange, and accumulate resistance genes embedded in gene cassettes (Gillings, 2014; Jechalke et al., 2014b), were frequently found on plasmids of the IncP-1ε group (Heuer et al., 2012). These plasmids are able to efficiently transfer and replicate in a broad range of hosts and are widely spread in clinical and environmental settings, such as agricultural soil and wastewater, where they might be important vectors of antibiotic and heavy metal resistance genes (Schlüter et al., 2007; Sen et al., 2011; Heuer et al., 2012; Popowska and Krawczyk-Balska, 2013).

However, in this study no significant positive correlations between relative abundance of resistance genes, class 1 integrons, IncP-1 plasmids and years of irrigation were observed, indicating no enrichment in the soil bacterial community. Similarly, no positive correlations were observed between the relative abundance of genes and total or bioaccessible concentrations of heavy metals or total and CaCl_2_ extracted concentrations of antibiotics (obtained from Supporting Information of Dalkmann et al., 2012, data not shown). Nevertheless, except for *qacE*+*qacEΔ1* the absolute and relative abundances of the detected resistance genes, class 1 integrons and IncP-1 plasmids were below the detection limit in the soils from rain-fed fields, i.e., soils never irrigated with wastewater, but were detectable in irrigated soils, pointing to the wastewater irrigation as a common source of these genes in soil. The large absolute numbers of *intI1, qacE+qacEΔ1*, and *aadA* genes in soils irrigated for 3 and 6 years could eventually be due to applications of cattle manure to these fields, since both fields show a relatively large TOC content (more similar to that of long-term wastewater-irrigated fields, Table S2). Duration of irrigation is for sure not longer than 3 and 6 years, since the wastewater distribution channel was constructed recently. However, it cannot be excluded that a few farmers applied manure irregularly on rain-fed fields before wastewater was available as fertilizer.

The abundances of *tet*(W) and *tet*(Q) were below detection limit in non-irrigated soils, which is in agreement with a previous study reporting low abundances of *tet*(W) and *tet*(Q) in agricultural soils without manure fertilization (Kyselková et al., 2013), and still close to the detection limit after irrigation, which might indicate a low concentration of these genes and their hosts in the irrigation water.

Positive correlations were observed between absolute abundances of *korB, tet*(W), and the bioaccessible concentration of Cu (Table 4), which further supports a common source of IncP-1 plasmids, tetracycline resistance genes, and this heavy metal. Correlations between absolute abundances of *korB, tet*(W), and Zn were significant for the total concentrations of this heavy metal but not for its bioaccessible fraction, which might indicate a less relevant association compared to Cu.

Not much data are available regarding minimum co-selective concentrations (MCCs) of heavy metals in soil, that potentially drive co-selection of antibiotic resistance (Seiler and Berendonk, 2012). In a field study of Berg et al. (2005), a concentration of 116.7 mg Cu/kg soil was associated with an increase of the absolute number of antibiotic resistant bacteria. The maximum concentration of 70 mg acid extractable Cu/kg soil detected in the Mexican soils was smaller than the one observed by Berg and colleagues, which might explain that no enrichment of resistance genes, IncP-1 plasmids and class 1 integrons occurred in the soil bacterial community over the period of 100 years of irrigation.

MCCs for Zn ranged from 19.61 μg/L in water samples to 22.75 mg/kg and 46.1 mg/kg in manure and sediment samples, respectively (Seiler and Berendonk, 2012), which is in the range of 46–281 mg Zn/kg detected in the Mexican soil samples but even lower than the 800 μg/L measured in wastewater samples from the Mezquital Valley (Guedron et al., 2014). This might indicate that higher concentrations of Zn are necessary in soil to co-select for antibiotic resistance. In accordance with this, Knapp et al. (2011) did not observe an increased abundance of antibiotic resistance genes in correlation with elevated Zn concentrations in archived Scottish soils, and similar to the results of the present study no positive correlations were found between metal concentrations and *tet*(Q) relative abundance. However, similar to the present study, Knapp et al. (2011) found correlations between Ni and Cu concentrations and *tet*(W) relative abundance.

Confirming a previous study by Gaze et al. (2011) for pig slurry, but in contrast to a study by Jechalke et al. (2014b) for bulk soil and rhizosphere, the abundance of *qacE* and *qacEΔ1* in irrigated Mexican soils were about three times higher than of *intI1* (Tukey test, *p* < 0.05), suggesting that more than one copy of these genes was associated with class 1 integrons or that these genes are carried by other integron classes, MGEs, or located chromosomally. The mean abundances of *korB, intI1*, and *aadA* genes were not significantly different, which might suggest that class 1 integrons and the streptomycin resistance gene *aadA* were associated with IncP-1 plasmids.

Besides changes in metal concentrations, a significant increase was also observed for S and P concentrations along with extended duration of irrigation. Although Shi et al. (2013) could show by a modeling approach that soil concentrations of S and P affected bacterial Cu resistance, in this study positive correlations between concentrations of S and P and antibiotic resistance levels were only observed for the absolute but not for the relative abundance, suggesting that there was no co-selection of antibiotic resistance genes at present S and P concentrations.

In summary, wastewater irrigation in Mezquital Valley, Mexico, over a period of 100 years led to an increase in bacterial biomass in soil and, accordingly, to an increase in absolute abundance of *intI1, qacE*+*qacEΔ1, aadA, korB*, and *tet*(W) genes. In parallel, concentrations of Zn, Cu, Pb, Ni, Cr, P, and S significantly increased. However, no significant positive correlations were observed between the relative abundance of selected genes and years of irrigation or concentration of heavy metals, indicating no enrichment in the soil bacterial community due to wastewater irrigation or due to a potential selection by increasing concentrations of heavy metals and antibiotic compounds. Nevertheless, the increase in gene copy numbers per gram of soil along with extended duration of irrigation might lead to a higher exposure of field crops and farmers to resistant bacteria, resistance genes and MGEs. Further epidemiological surveys are needed to address if the increased presence of antibiotic resistance genes in the soils of this region poses a health risk for the inhabitants of the region and the consumers of the crops.

### Conflict of interest statement

The authors declare that the research was conducted in the absence of any commercial or financial relationships that could be construed as a potential conflict of interest.
