# Supplementary material for: Effects of 100 years wastewater irrigation on resistance genes, class 1 integrons and IncP-1 plasmids in Mexican soil
Source: Front Microbiol. 2015 Mar 3;6:163. doi: 10.3389/fmicb.2015.00163 (PMC4347510; doi:10.3389/fmicb.2015.00163)
Supplement: Supplementary file 1 [file Table1.DOCX]

**Table S1:** Total concentrations of metals and inorganic compounds within the chronosequence.

| **years** | **Zn [mg/g]** | **Cu [mg/g]** | **Pb [mg/g]** | **Ni [mg/g]** | **Cr [mg/g]** | **Mn [mg/g]** | **P [mg/g]** | **S [mg/g]** |
| --- | --- | --- | --- | --- | --- | --- | --- | --- |
| 0a | 0.05 | 0.01 | nd | 0.02 | 0.04 | 0.79 | 0.48 | 0.18 |
| 0b | 0.05 | 0.01 | nd | 0.02 | 0.04 | 0.76 | 0.53 | 0.23 |
| 1.5 | 0.06 | 0.01 | nd | 0.02 | 0.04 | 0.59 | 0.49 | 0.35 |
| 3a | 0.06 | 0.01 | nd | 0.02 | 0.03 | 0.67 | 0.71 | 0.81 |
| 3b | 0.05 | 0.01 | nd | 0.02 | 0.04 | 0.72 | 0.79 | 0.26 |
| 6 | 0.06 | 0.01 | 0.03 | 0.02 | 0.04 | 0.73 | 0.50 | 0.27 |
| 8 | 0.05 | 0.02 | nd | 0.02 | 0.03 | 0.76 | 0.30 | 0.21 |
| 85 | 0.23 | 0.06 | 0.06 | 0.04 | 0.10 | 0.66 | 1.10 | 0.65 |
| 100a | 0.18 | 0.05 | 0.03 | 0.03 | 0.06 | 0.51 | 1.48 | 0.87 |
| 100b | 0.28 | 0.07 | 0.06 | 0.04 | 0.10 | 0.57 | 1.87 | 1.12 |

nd = not detected
