# Supplementary material for: Effects of 100 years wastewater irrigation on resistance genes, class 1 integrons and IncP-1 plasmids in Mexican soil
Source: Front Microbiol. 2015 Mar 3;6:163. doi: 10.3389/fmicb.2015.00163 (PMC4347510; doi:10.3389/fmicb.2015.00163)
Supplement: Supplementary file 2 [file Table2.DOCX]

**Table S2:** Bioaccessible concentrations of metals, phosphorus as well as electrical conductivity (E.c.), pH and total organic carbon (TOC) within the chronosequence.

| **years** | **Zn [µg/g]** | **Cu [µg/g]** | **Cd [ng/g]** | **P [mg/g]** | **E.c. [µS/cm]** | **pH** | **TOC [g/kg]** |
| --- | --- | --- | --- | --- | --- | --- | --- |
| 0a | 0.02 | 0.03 | 0.01 | 0.04 | 53 | 6.3 | 9.10 |
| 0b | na | na | na | na | na | na | na |
| 1.5 | na | na | na | na | 254 | 6.7 | 10.60 |
| 3a | 0.03 | 0.03 | 0.08 | na | 2020 | 6.6 | 19.40 |
| 3b | 0.04 | 0.01 | 0.20 | na | 166 | 6.4 | 13.50 |
| 6 | na | na | na | na | 224 | 7.0 | 16.20 |
| 8 | 0.05 | 0.01 | 0.48 | 0.08 | 219 | 6.7 | 11.60 |
| 85 | 0.08 | 0.11 | 6.50 | 0.04 | 198 | 6.7 | 20.60 |
| 100a | 0.08 | 0.08 | 4.85 | 0.03 | 275 | 6.9 | 31.50 |
| 100b | 0.07 | 0.11 | 4.06 | 0.05 | 242 | 6.8 | 33.50 |

na = not analyzed
